# Development and Immunogenicity Evaluation of an RSV Recombinant Vaccine Displaying a Conserved Domain of RSV G

**DOI:** 10.3390/vaccines14040311

**Published:** 2026-03-30

**Authors:** Jingjing Ma, Xinjie Wang, Shijia Li, Zhibin Li, Fei Wang, Yu Zhang, Lingyun Li, Junli Jia, Huamin Tang

**Affiliations:** 1 Department of Immunology, Nanjing Medical University, Nanjing 211166, China; majingjing0615@stu.njmu.edu.cn (J.M.); 2024110109@stu.njmu.edu.cn (S.L.); 2025210033@stu.njmu.edu.cn (Z.L.); 2024110123@stu.njmu.edu.cn (Y.Z.); 2 School of Basic Medical Sciences, Nanjing Medical University, Nanjing 211166, China; 22220219@stu.njmu.edu.cn; 3 Department of Medical Genetics, Nanjing Medical University, Nanjing 211166, China; lilingyun@njmu.edu.cn; 4 The Laboratory Center for Basic Medical Sciences, Nanjing Medical University, Nanjing 211166, China; 5 Department of Immunology, National Vaccine Innovation Platform, School of Basic Medical Sciences, Nanjing Medical University, Nanjing 211166, China

**Keywords:** RSV, HBsAg, SVPs, G protein, vaccine, immunogenicity

## Abstract

Background: Respiratory syncytial virus (RSV) causes severe lung infections in infants and the elderly. The conserved central domain (CCD) of the RSV G protein is a key antigenic fragment for inducing protective antibodies. In this study, we used the hepatitis B surface antigen (HBsAg) as a platform to present this RSV G CCD fragment. Methods: We first sequenced and compared several HBsAg genotypes from clinical samples and selected one as an expression candidate for further development. The RSV G CCD was then inserted into the selected candidate to generate a recombinant expression construct. Subviral particles (SVPs) were produced using both CHO cells and yeast expression systems. Particle assembly was examined using electron microscopy. Finally, the safety and immunogenicity of the recombinant vaccine were evaluated in mice. Results: We successfully identified HBsAg38 as a potential recombinant vaccine expression candidate due to its abundant expression and secretion. The RSV G CCD fragment was inserted into the candidate and efficiently expressed in both CHO cells and yeast. The expressed protein was effectively secreted and formed uniform, spherical particles. The resulting vaccine candidate was safe for mice, causing no detectable weight loss or organ damage. Immunization with the recombinant SVPs elicited antibody responses against both HBsAg and the RSV G CCD. Upon intranasal RSV challenge, vaccinated mice exhibited markedly reduced RSV F protein and mRNA levels in lung tissues compared to PBS controls, with the yeast-derived SVP group showing the most pronounced reduction. Histopathological analysis further revealed that immunized mice had significantly less alveolar destruction and inflammatory cell infiltration than the control group, confirming that the vaccine conferred effective protection against RSV-induced lung pathology. Conclusions: We successfully developed a novel antigen-displaying HBsAg platform for generating vaccines targeting multiple pathogens. The RSV G CCD-expressing HBsAg induced a strong antibody response and provided effective protection against RSV infection. This platform offers a promising new approach for the development of next-generation vaccines.

## 1. Introduction

Respiratory syncytial virus (RSV) is a leading cause of acute lower respiratory tract infections (LRTI) worldwide, resulting in severe illnesses such as bronchiolitis and pneumonia, particularly in infants, the elderly, and immunocompromised individuals. A global analysis by Li et al. [1] estimated that in 2019, RSV was associated with approximately 33 million cases of acute LRTI and 3 million hospitalizations worldwide, underscoring its substantial burden on healthcare systems.

The RSV life cycle relies on two key surface proteins for host cell entry. The fusion (F) protein mediates fusion of the viral envelope with the host cell membrane, a critical step for viral entry [2]. The attachment (G) protein facilitates viral attachment by interacting with cellular receptors [3]. Due to their essential roles in infection, both F and G proteins have been prioritized as key antigens in RSV vaccine development [4].

Historically, the F protein has been the primary focus of vaccine research as the main target of neutralizing antibodies, with its conformational state being a critical determinant of immunogenicity [5]. Early vaccine attempts using formalin-inactivated RSV (FI-RSV) resulted in enhanced respiratory disease upon natural infection [6,7]. This aberrant response was later attributed to the disruption of protective F protein epitopes during inactivation, leading to the induction of low-affinity, non-neutralizing antibodies and a pathological Th2-biased immune response [7,8,9]. In recent years, significant progress has been made in stabilizing the prefusion conformation of the F protein, culminating in FDA-approved vaccines such as GSK’s Arexvy and Pfizer’s Abrysvo for older adults and maternal immunization, as well as Moderna’s mRESVIA (mRNA-1345), an mRNA-based vaccine encoding the prefusion F protein [10,11,12]. In the area of passive immunoprophylaxis, long-acting monoclonal antibodies, including nirsevimab and the recently approved clesrovimab, have further expanded the preventive options for infants [13,14]. Despite these advances, current prefusion F-based vaccines are not approved for direct use in young children, and emerging evidence indicates that vaccine efficacy in older adults may decline over successive RSV seasons [15]. Furthermore, the inherent structural instability of the F protein can pose challenges for its incorporation into multivalent or complex fusion-antigen designs, potentially limiting platform flexibility.

These challenges have renewed interest in the RSV G protein as a vaccine target. The central conserved domain (CCD) of the G protein is highly conserved across RSV subtypes A and B [4]. This domain facilitates viral attachment by interacting with the CX3CR1 receptor on human respiratory epithelial cells and is a target for broadly neutralizing antibodies [16,17]. Numerous studies have demonstrated that immunity directed against the G protein CCD can elicit specific antibody responses and contribute to protection against RSV infection [18,19,20]. Importantly, as a key neutralizing epitope, the CCD is less prone to inducing non-neutralizing antibodies, thereby potentially reducing the risk of vaccine-enhanced disease (VED) [21].

Virus-like particles (VLPs) offer a compelling platform for antigen presentation by mimicking the native architecture of viruses without incorporating genetic material [20]. VLPs are non-infectious, self-assembling nanostructures typically 20–200 nm in diameter, classified into non-enveloped types (e.g., bacteriophage Qβ, HPV L1, HBcAg) and enveloped types (e.g., HBsAg SVPs, influenza VLPs) [22]. Several VLP-based vaccines have been licensed, including Engerix-B and Recombivax HB against HBV, Gardasil 9 and Cervarix against HPV, Hecolin against HEV, and Mosquirix against malaria [23]. The hepatitis B surface antigen (HBsAg) is one of the most extensively validated VLP platforms. It self-assembles into highly immunogenic subviral particles (SVPs) and has a proven track record of safety and efficacy in licensed vaccines [24,25]. Critically, HBsAg SVPs can accommodate foreign antigens, displaying exogenous epitopes on their surface through genetic fusion or insertion. The success of the RTS, S/AS01 malaria vaccine, which fuses a malaria antigen with HBsAg to form chimeric SVPs, exemplifies the potential of this approach [26,27]. Similarly, insertion of the preS1 domain from hepatitis B virus into HBsAg yielded chimeric particles that retained assembly capability and induced robust antibody responses against both targets in preclinical models [28]. These studies confirm that the HBsAg platform can be adapted to present foreign antigens while maintaining its structural and immunological integrity. However, this platform has not yet been explored for RSV vaccine development, which prompted us to investigate its potential in this context. To this end, we evaluated the advantages of HBsAg relative to other available VLP systems. Compared to other VLP platforms such as those based on bacteriophage, ferritin nanoparticles, or Newcastle disease virus, the HBsAg SVP platform offers several distinct advantages: (i) an unparalleled clinical safety record across all age groups, including neonates; (ii) clinical proof-of-concept as a chimeric antigen carrier; and (iii) compatibility with both yeast and mammalian cell expression systems, facilitating scalable manufacturing.

Although the HBsAg SVP platform has been widely used in licensed vaccines, existing commercial sequences often represent a limited subset of viral genotypes. Given the sequence diversity of HBsAg in clinical settings, we reasoned that selecting an expression-optimized variant from patient-derived isolates could further enhance the manufacturability and immunogenicity of the chimeric vaccine. To this end, we first conducted a comparative analysis of several clinically derived HBsAg genotypes to identify a candidate with superior secretion efficiency as the carrier for the RSV G CCD.

Based on these findings, we developed a recombinant vaccine candidate by inserting the CCD (amino acids 159–191) of the RSV G protein into the selected HBsAg carrier. This design aims to exploit the intrinsic assembly properties of HBsAg to generate chimeric SVPs displaying the RSV G CCD on their surface. By presenting both antigens in a single particulate structure, this strategy is intended to elicit simultaneous humoral immune responses against HBsAg and the RSV G protein. More broadly, this approach offers a versatile platform for the development of RSV vaccines and highlights the potential of established VLP platforms for designing multivalent vaccines against pathogens.

## 2. Materials and Methods

### 2.1. Cell Culture and RSV Propagation

HEK293T, CHO, and Hep2 cells were obtained from authenticated cell banks and maintained in Dulbecco’s modified Eagle’s medium (DMEM) supplemented with 10% fetal bovine serum (FBS). All cells were cultured at 37 °C in a humidified incubator with 5% CO_2_.

The RSV A2 strain was propagated in Hep2 cells. Upon the appearance of extensive cytopathic effects (CPE) reaching approximately 70–80% confluence, both infected cells and culture supernatants were harvested and centrifuged at 2000× *g* for 10 min to separate the cellular fraction. The supernatant was collected, while the cell pellet was resuspended in 1 mL of culture medium, followed by repeated freeze–thaw cycles using liquid nitrogen to release intracellular virus. The lysate was then centrifuged again at 2000× *g* for 10 min to remove cell debris. The resulting supernatant was combined with the previously collected fraction. To concentrate the virus, the mixture was layered onto a 20% (*w*/*v*) sucrose cushion and ultracentrifuged at 150,000× *g* for 5 h. The virus pellet was resuspended in PBS to achieve a 1000-fold concentration. Virus stocks were aliquoted and stored at −80 °C for future use.

### 2.2. Plasmid Construction

To construct the HBsAg expression plasmid, total RNA was extracted from peripheral blood samples of patients with HBV infection and reverse-transcribed into cDNA. The HBsAg coding sequence was then amplified by PCR using specific primers (forward: 5′-acagaattcgcaccgaacatggaga-3′; reverse: 5′-acactcgagttaaatgtatacccaaagac-3′) that introduced EcoRI and XhoI restriction sites. After dual digestion with EcoRI and XhoI, the amplified fragment was ligated into the pCAGGS vector that had been digested with the same enzymes to generate the pCAGGS-HBsAg plasmid.

To generate the HBsAg-RSV G CCD recombinant construct, the CCD of the RSV G protein was inserted between amino acids 126 and 127 of the HBsAg protein. This was accomplished by site-directed insertion using overlap extension PCR with the pCAGGS-HBsAg plasmid as the template. The resulting full-length chimeric fragment was then cloned into the pCAGGS vector, yielding the final construct, pCAGGS-HBsAg-RSV G CCD.

For yeast-based expression, the HBsAg-RSV G CCD recombinant sequence was amplified from pCAGGS-HBsAg-RSV G CCD. The PCR product was then digested and cloned into the pPIC9K vector, generating the pPIC9K-HBsAg-RSV G CCD expression plasmid.

To evaluate the impact of specific amino acid substitutions on recombinant protein expression and SVP assembly, site-directed mutagenesis was performed using pCAGGS-HBsAg53 as the parental template. A two-step overlap extension PCR approach was employed. In the first round, two separate PCR reactions were performed using mutagenic primers paired with flanking primers to generate overlapping fragments containing the desired point mutation. In the second round, the two overlapping fragments were combined and amplified using the flanking primers alone to produce the full-length mutated sequence. Single-point mutants (N3S and T126I), along with the corresponding double mutant (N3S/T126I), were engineered. The resulting constructs were designated as pCAGGS-HBsAg53-N3S, pCAGGS-HBsAg53-T126I, and pCAGGS-HBsAg53-N3S/T126I, respectively.

### 2.3. Antibodies

To obtain specific antibodies against HBsAg, mice were immunized via footpad injection. First, HEK293T cells were transfected with the pCAGGS-HBsAg expression plasmid using polyethyleneimine (PEI, Polysciences) according to the manufacturer’s instructions. After 72 h, the culture supernatant was collected and centrifuged at low speed to remove cells and debris. The supernatant was then subjected to ultracentrifugation (150,000× *g* for 5 h) over a 20% sucrose cushion to enrich for SVPs formed by HBsAg, which were used as the immunogen. Following three rounds of immunization, mouse serum was collected for subsequent Western blot and immunoassay analyses.

Anti-RSV F protein antibody was purchased from Santa Cruz Biotechnology (SC-101362) and used according to the manufacturer’s instructions.

### 2.4. Expression and Purification of HBsAg–RSV G CCD Recombinant SVPs

#### 2.4.1. Expression and SVP Purification in CHO Cells

To evaluate expression in mammalian systems, CHO cells were transfected with the pCAGGS-HBsAg-RSV G CCD and subjected to puromycin selection to establish a stable cell line. Following clonal expansion, the culture supernatants were harvested and clarified via low-speed centrifugation to eliminate cellular debris. To isolate and enrich the recombinant SVPs, the clarified supernatants were subjected to ultracentrifugation through a 20% (*w*/*v*) sucrose cushion.

#### 2.4.2. Expression and SVP Purification in *Pichia pastoris* GS115

The pPIC9K-HBsAg-RSV G CCD expression vector was linearized and electroporated into the GS115 strain of *P. pastoris*. Following electroporation, positive clones were selected on YPD agar containing Geneticin (G418), as the vector conferred resistance to this antibiotic. Selected clones were initially cultured in Buffered Glycerol-complex Medium (BMGY) to achieve sufficient biomass. For protein induction, the cells were transferred to Buffered Methanol-complex Medium (BMMY), where expression was maintained over 72 h through daily methanol supplementation. The resulting culture supernatants were processed using the same clarification and sucrose cushion ultracentrifugation protocols employed for the CHO cell cultures to ensure consistency in SVP enrichment across both platforms.

### 2.5. Western Blot Analysis

To assess protein expression, cells were collected and lysed in RIPA buffer (Beyotime). The resulting protein lysates were resolved by SDS-PAGE and subsequently transferred onto polyvinylidene difluoride (PVDF) membranes. After transfer, membranes were blocked to reduce nonspecific binding and incubated with the primary antibodies. After a series of washes, the membranes were incubated with HRP-conjugated secondary antibodies. Protein signals were detected using enhanced chemiluminescence (ECL) reagents. Images were acquired with an Amersham ImageQuant 600 imaging system (GE Healthcare, Chicago, IL, USA).

### 2.6. Transmission Electron Microscopy (TEM) Analysis

The morphological integrity and size distribution of the purified HBsAg-RSV G CCD SVPs were characterized using negative-stain transmission electron microscopy. Briefly, the purified samples were diluted to an appropriate concentration and applied onto carbon-coated copper grids (200 mesh). After allowing the particles to adsorb for several minutes, the grids were negatively stained with uranyl acetate to enhance structural contrast. Excess stain was carefully blotted with filter paper, and the grids were air-dried at room temperature. The specimens were examined using a JEM-1400 Flash transmission electron microscope (JEOL, Tokyo, Japan). Representative micrographs were captured to evaluate the homogeneity, overall morphology, and assembly efficiency of the recombinant protein particles.

### 2.7. Evaluation of Immunogenicity of HBsAg-RSV G CCD SVPs in C57BL/6 Mice

To evaluate the immunogenicity of the recombinant proteins, 6~8-week-old female C57BL/6 mice were employed. The mice were randomly assigned to three experimental groups: a PBS-mock control group, a CHO-derived SVP group (SVP-C), and a Yeast-derived SVP group (SVP-Y). The vaccination followed a three-dose regimen, consisting of a primary immunization on Day 0, followed by two booster injections on Day 14 and Day 28. For vaccine formulation, the purified SVPs were blended with an aluminum hydroxide adjuvant (Alum, InvivoGen, San Diego, CA, USA). Each mouse was administered the assigned formulation via intraperitoneal (i.p.) injection. Serum samples were collected via retro-orbital puncture at 14 days after each immunization (Day 14, Day 28, and Day 42, respectively). The collected blood was allowed to clot at room temperature and subsequently centrifuged to isolate the sera, which were stored at −80 °C for downstream antibody titer quantification. The sera collected on Day 42 (14 days after the final boost) were used for antibody titer determination by IFA (Indirect immunofluorescence assay).

### 2.8. Indirect Immunofluorescence Assay (IFA)

Specific antibody responses against HBsAg and RSV G CCD were evaluated by IFA using antigen-expressing cells. Cells expressing the indicated antigens were fixed and permeabilized, followed by incubation with serial dilutions of mouse sera. After washing, the cells were incubated with a FITC-conjugated goat anti-mouse IgG secondary antibody. Cell nuclei were counterstained with DAPI. Fluorescent signals were examined under a fluorescence microscope. Antibody titers were defined as the reciprocal of the highest serum dilution that produced a clear and specific fluorescent signal above the background observed in negative control samples.

### 2.9. RSV Challenge Experiment in Mice

To assess the protective efficacy of the vaccine candidates, immunized mice were challenged with RSV on Day 42 of the study, corresponding to two weeks after the final booster immunization. Mice were anesthetized by intraperitoneal injection of urethane and intranasally inoculated with RSV at a dose of 1 × 10^6^ PFU per mouse in a total volume of 50 μL [29]. On day 4 post-infection, corresponding to the peak of viral replication, mice were euthanized for tissue collection. Lung tissues were harvested and divided for downstream analyses. One portion was used for viral protein detection and quantitative RT-PCR analysis of viral mRNA levels, while the remaining tissue was fixed in 4% paraformaldehyde for subsequent histopathological examination.

### 2.10. RSV Neutralization Assay

To assess the neutralizing activity of vaccine-induced antibodies, an RSV neutralization assay was performed using GFP-expressing recombinant RSV (RSV-GFP) [30]. Sera were collected from immunized mice after the third immunization and diluted to 1:50. The diluted sera were incubated with RSV-GFP at 37 °C for 1 h to allow antibody-virus binding. The virus-serum mixtures were then transferred onto Hep2 cells and incubated at 37 °C with 5% CO_2_ for 48 h. GFP fluorescence was observed under a fluorescence microscope to evaluate the neutralizing activity of the immune sera. All assays were performed in triplicate.

### 2.11. Quantitative Real-Time RT-PCR Analysis of RSV F Gene Expression

Quantitative analysis of *RSV F* gene expression was performed by real-time RT-PCR using SYBR Green chemistry. Total RNA was extracted from lung homogenates using TRIzol Reagent (TIANGEN, Beijing, China) and reverse-transcribed into cDNA. Real-time PCR was then carried out on an ABI StepOne Plus Real-Time PCR System (Applied Biosystems, Carlsbad and Foster City, CA, USA) with TB Green Premix Ex Taq (TaKaRa, San Jose, CA, USA). The primers used for *RSV F* gene amplification were: forward, 5′-CGAGCCAGAAGAGAACTACCA-3′; reverse, 5′-CCTTCTAGGTGCAGGACCTTA-3′. Thermal cycling conditions were set according to the manufacturer’s recommended protocol. Relative quantification of RSV mRNA was calculated using the 2^−ΔΔCt method, with β-actin as the internal reference gene. All samples were analyzed in triplicate.

### 2.12. Hematoxylin and Eosin (H&E) Staining

Lung tissues were fixed in 4% paraformaldehyde, embedded in paraffin, and sectioned at 4–5 μm thickness. Sections were deparaffinized in xylene, rehydrated through graded ethanol, and stained with hematoxylin for 10 min. After rinsing, sections were counterstained with eosin for 30 s to 2 min, then dehydrated and mounted. Histopathological changes were observed under a light microscope.

### 2.13. Quantification of HBsAg Secretion by ELISA

HBsAg levels in cell culture supernatants were quantified by Enzyme-Linked Immunosorbent Assay (ELISA). Briefly, 96-well plates were coated with anti-HBsAg capture antibody overnight at 4 °C. After blocking with 3% BSA, supernatants were added and incubated for 2 h at 37 °C, followed by HRP-conjugated anti-HBsAg detection antibody for 1 h at 37 °C. TMB substrate was added, and the reaction was stopped with 2 M H_2_SO_4_. Absorbance was read at 450 nm. HBsAg concentrations were calculated from a standard curve.

### 2.14. Statistical Analysis

All data are presented as mean ± standard deviation (SD). Statistical analyses were performed using one-way analysis of variance (ANOVA) to compare differences among experimental groups. All analyses were conducted using GraphPad Prism 6.0 (GraphPad Software, San Diego, CA, USA). *p*-value < 0.05 was considered statistically significant.

### 2.15. Ethical Statement

All animal experiments were conducted in accordance with the Regulations for the Administration of Affairs Concerning Experimental Animals of China. The study protocols were reviewed and approved by the Institutional Animal Care and Use Committee (IACUC) of Nanjing Medical University (Approval No. 2411001). Efforts were made to minimize the number of animals used and to reduce animal discomfort throughout the study.

## 3. Results

### 3.1. Identified HBsAg38 as an Efficient Expression Platform

**Figure 1:**
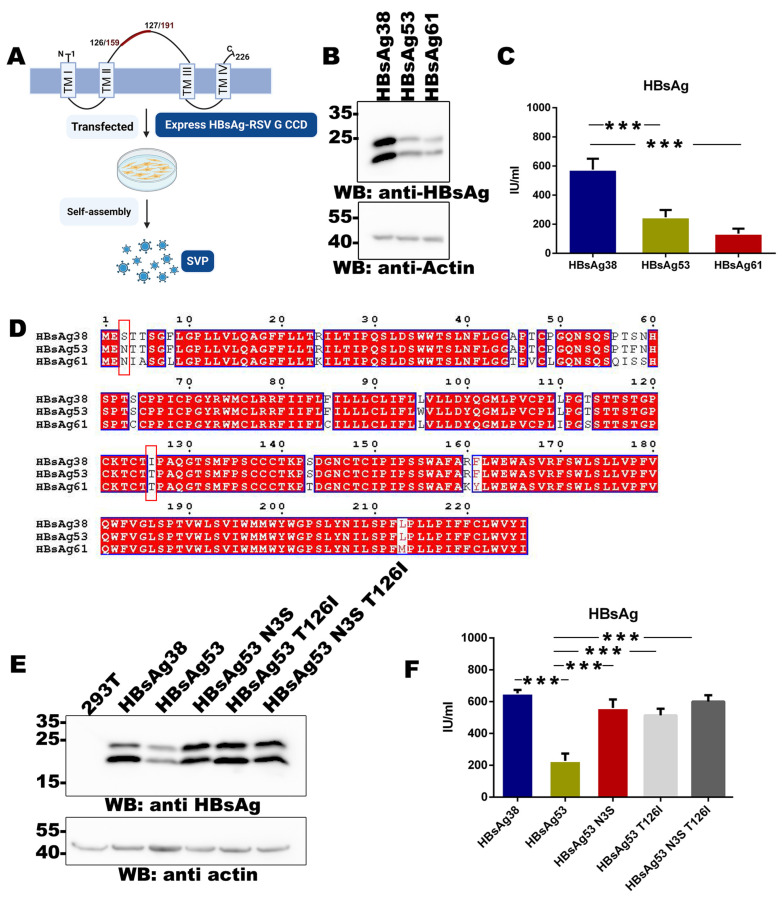
Screening and optimization of HBsAg variants as scaffolds for SVP assembly: (**A**) Schematic representation of the HBsAg-based SVP presentation platform. (**B**) Western blot (WB) analysis of HBsAg expression in transiently transfected cells. HBsAg38, HBsAg53, and HBsAg61 were detected using an anti-HBsAg antibody (**top**), and the loading control was assessed with an anti-actin antibody (**bottom**). (**C**) ELISA quantification of HBsAg secreted into the culture supernatant from the indicated isolates. Data are presented as mean ± SD. *** *p* < 0.001. (**D**) Amino acid sequence alignment of HBsAg38, HBsAg53, and HBsAg61. Red boxes highlight the key residue differences at positions 3 and 126. (**E**,**F**) Validation of site-directed mutagenesis (N3S, T126I) on HBsAg53 expression and secretion. Intracellular protein levels were detected by Western blot (**E**), and supernatant secretion levels were measured by ELISA (**F**). Data are presented as mean ± SD. *** *p* < 0.001.

To establish an HBsAg-based VLP presentation system for RSV G CCD expression, we first screened a range of HBsAg sequences derived from various clinical sources. Specifically, total RNA was extracted from peripheral blood samples of 20 HBV-infected patients and reverse-transcribed into cDNA. The HBsAg coding sequences were amplified by PCR, of which 15 were successfully cloned. Sequencing analysis identified three distinct amino acid sequence variants, designated HBsAg38, HBsAg53, and HBsAg61 (a sequence alignment of all variants is provided in Supplementary Materials). It has been well-established that HBsAg can self-assemble into virus-like particles (SVPs) when expressed in mammalian cells, a characteristic that makes it an ideal platform for displaying foreign antigens (Figure 1A). Leveraging this characteristic, we evaluated these three HBsAg isolates by comparing their expression and secretion profiles to identify the optimal candidate for use in RSV G CCD expression. The three HBsAg variants were transiently expressed in HEK293T cells. Western blot and ELISA analyses revealed significant variations in expression and secretion levels among the candidates. Notably, the HBsAg38 variant exhibited the highest intracellular expression (Figure 1B) and the most robust secretory capacity into the culture supernatant (Figure 1C), making it a promising candidate for further evaluation.

In an effort to elucidate the molecular basis of these differences, amino acid sequences of HBsAg38, HBsAg53, and HBsAg61 were aligned (Figure 1D). The alignment demonstrated high overall sequence conservation; nevertheless, two unique substitutions were identified at positions 3 and 126, where HBsAg38 diverged from both HBsAg53 and HBsAg61.

The impact of these residues was validated by introducing N3S and T126I mutations into the HBsAg53 backbone. Western blot analysis confirmed that while wild-type HBsAg53 showed weaker intracellular expression than HBsAg38, the introduction of either N3S or T126I mutations led to a marked recovery in expression levels (Figure 1E). ELISA quantification of culture supernatants further demonstrated that both single mutations and the double mutation (N3S/T126I) significantly enhanced HBsAg secretion efficiency (Figure 1F). These findings indicate that the residues at positions 3 and 126 are critical determinants of HBsAg expression and secretion, and that the S3 and I126 residues present in HBsAg38 contribute to its superior performance as an expression platform.

### 3.2. RSV G CCD Is Efficiently Expressed on the HBsAg38 Platform

**Figure 2:**
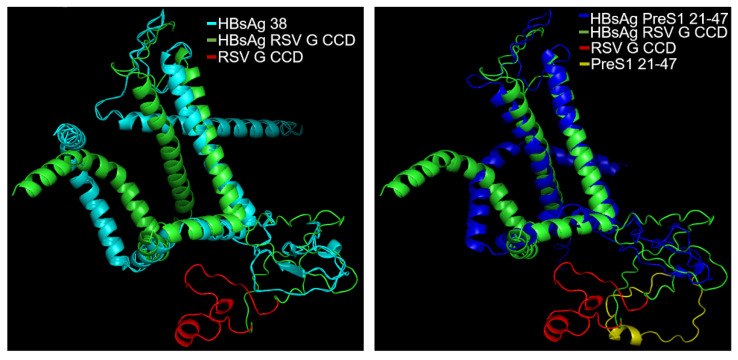
Structural modeling of the HBsAg38–RSV G CCD recombinant protein. (**Left**) AlphaFold3-predicted structure of the HBsAg38–RSV G CCD recombinant protein (green/red) superimposed with the wild-type HBsAg38 scaffold (cyan). (**Right**) Structural comparison between the HBsAg–RSV G CCD recombinant model and a chimeric HBsAg model carrying the HBV PreS1 domain (blue/yellow) inserted at the 126–127 locus. The RSV G CCD (red) is predicted to be outwardly exposed on the external antigenic loop of the SVP.

Based on the screening results, HBsAg38 was selected as the recombinant scaffold for RSV G CCD. To assess the structural impact of the foreign fragment insertion, we performed protein structure modeling of the HBsAg38-RSV G CCD recombinant protein (Figure 2). AlphaFold3 predictions demonstrated that the core architecture of HBsAg—including its critical transmembrane helices—remained undisturbed following the recombinant. Notably, the global conformation was closely analogous to that of the established chimeric HBsAg model in which the HBV PreS1 domain is inserted into the 126–127 amino acid locus [28]. Given that this specific site is a validated region known to accommodate insertions without abolishing SVP assembly, our model confirmed that the RSV G CCD was similarly positioned within this external antigenic loop. The predicted structure showed the RSV G CCD in an outwardly exposed orientation, fulfilling the design criteria for maximum epitope accessibility on the surface of the assembled SVPs.

### 3.3. Stable Expression and Self-Assembly of HBsAg-RSV G CCD SVPs Both in CHO and P. pastoris Expression Systems

**Figure 3:**
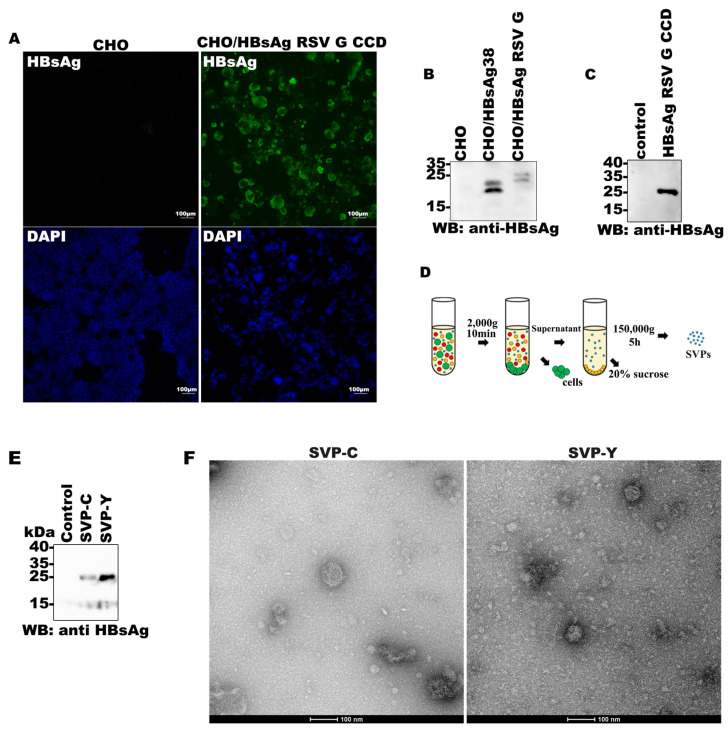
Expression and morphological characterization of HBsAg-RSV G CCD SVPs: (**A**) Immunofluorescence analysis of CHO cells transfected with either empty vector (CHO) or HBsAg-RSV G CCD. Cells were stained for HBsAg expression (green) and nuclear staining with DAPI (blue). (**B**,**C**) Western blot detection of HBsAg-RSV G CCD in CHO cell lysates (**B**) and *P. pastoris* culture supernatants (**C**). (**D**) Schematic of the SVP purification workflow using sucrose cushion enrichment. (**E**) Western blot analysis of purified SVPs from CHO and Yeast expression systems. (**F**) Negative-stain transmission electron microscopy (TEM) images showing the spherical morphology and size distribution (20–25 nm) of SVPs derived from Yeast (**left**) and CHO (**right**) platforms. Scale bars = 100 nm.

To evaluate the expression of the HBsAg-RSV G CCD recombinant protein across different systems, the construct was expressed both in CHO and *P. pastoris*. In mammalian cells, immunofluorescence confirmed the expression of the recombinant protein (Figure 3A), and Western blot analysis of cell lysates revealed a specific band consistent with the predicted molecular weight (Figure 3B). In the yeast system, Western blot analysis of culture supernatants identified a distinct band, demonstrating successful secretory expression of the recombinant protein (Figure 3C). Taken together, these results indicate that the HBsAg-RSV G CCD recombinant protein achieves consistent and substantial expression in both mainstream vaccine production platforms. This highlights the excellent structural tolerance of the HBsAg scaffold toward the RSV G CCD fragment, providing a robust technical foundation for scaled-up production and downstream immunogenicity evaluation.

To determine whether the HBsAg-RSV G CCD recombinant protein could self-assemble into SVPs, recombinant proteins were purified from both CHO and yeast culture supernatants. Following enrichment through a 20% sucrose cushion, the samples were collected and analyzed for the presence of HBsAg-RSV G CCD particles (Figure 3D). Western blot analysis of the concentrated fractions showed strong HBsAg-specific signals (Figure 3E). Furthermore, TEM revealed an abundance of regular, spherical particles in both samples. These particles exhibited a clear, well-defined morphology with diameters predominantly ranging from 20 to 25 nm (Figure 3F). Collectively, these findings demonstrate that the HBsAg-RSV G CCD recombinant protein successfully self-assembles into structurally homogenous virus-like particles regardless of the expression system utilized.

### 3.4. Recombinant HBsAg-RSV G CCD SVPs Are Safe in a Murine Model

**Figure 4:**
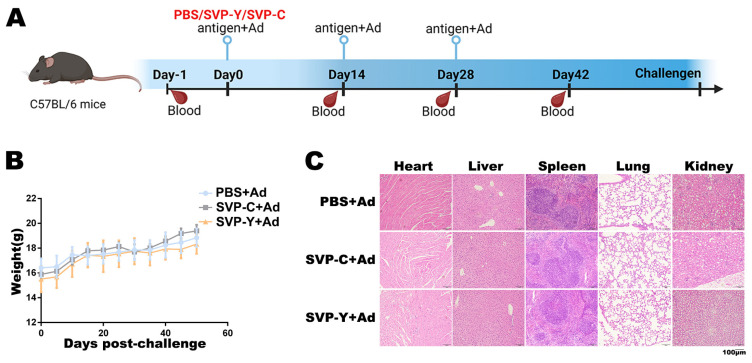
In vivo safety evaluation of HBsAg-RSV G CCD SVPs in mice: (**A**) Schematic of the immunization schedule in C57BL/6 mice, including the prime-boost regimen (prime on Day 0, boosters on Day 14 and Day 28; serum collected on Day 14, Day 28, and Day 42; RSV challenge on Day 42) and time points for blood collection and RSV challenge. (**B**) Dynamic changes in body weight of mice in the PBS, SVP-Y, and SVP-C groups. (**C**) Representative H&E-stained sections of major organs (heart, liver, spleen, lung, and kidney) obtained after completion of the immunization regimen, showing no evident tissue damage or inflammatory lesions.

C57BL/6 mice were immunized according to the established agenda (Figure 4A). Throughout the immunization and observation periods, mice in all groups exhibited a gradual increase in body weight over time. No significant differences were observed among the PBS control group, the SVP-Y group, and the SVP-C group (Figure 4B). During the immunization period, no reduction in activity, abnormal feeding behavior, or injection site reactions were observed in any of the groups.

Upon completion of the immunization schedule, histological observations were performed on the major organs of the mice. Heart, liver, spleen, lung, and kidney tissues were processed for H&E staining analysis. The results showed that the organs in all immunization groups possessed clear structures and normal tissue architecture. No significant inflammatory cell infiltration or tissue damage was observed across the groups, with morphologies similar to those of the PBS control group (Figure 4C).

These results indicate that under the dosage and immunization schedule employed in this study, the HBsAg-RSV G CCD recombinant SVPs exhibit a favorable safety profile in mice.

### 3.5. Recombinant HBsAg-RSV G CCD SVPs Induce Protective Immunity and Reduce Viral Load in Mice

**Figure 5:**
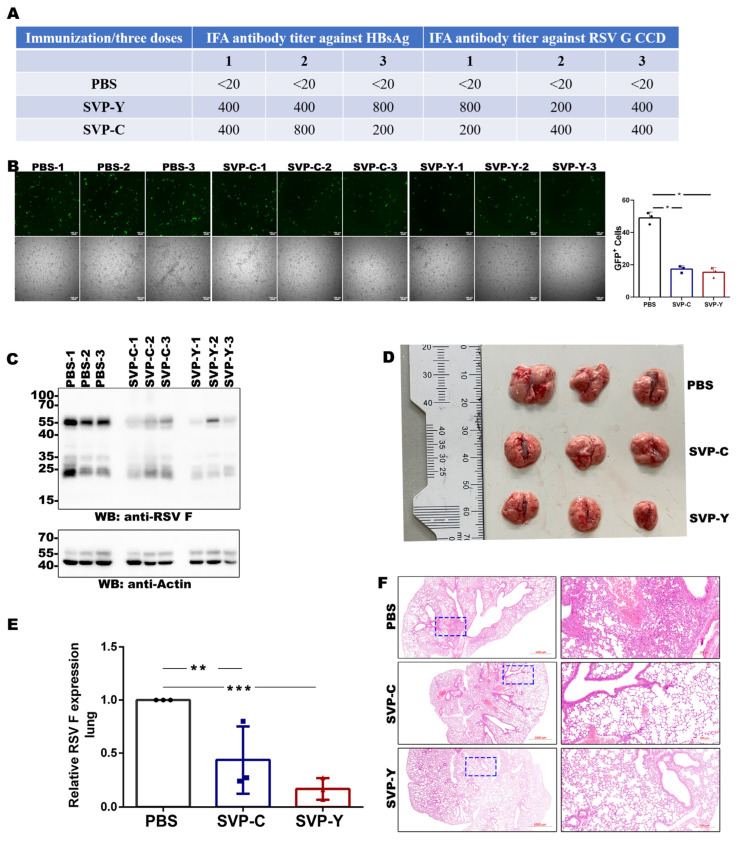
Protective efficacy and lung pathology following RSV challenge: (**A**) Table of IFA antibody titers against HBsAg and RSV G CCD in immunized mice (*n* = 3 per group). (**B**) RSV neutralization assay using RSV-GFP. Sera collected from mice after the third immunization were diluted at 1:50 and incubated with RSV-GFP before infection of Hep2 cells. After 48 h, GFP fluorescence (**upper panels**) and bright-field images (**lower panels**) were captured. The bar graph on the right shows the quantification of GFP-positive cells per field. Data are presented as mean ± SD. * *p* < 0.05, ** *p* < 0.01, *** *p* < 0.001. (**C**) Western blot analysis of RSV F protein levels in lung homogenates 4 days post-infection. (**D**) Gross morphological observation of harvested lungs, showing reduced hyperemia and swelling in immunized groups compared to the PBS control. (**E**) Relative expression of RSV F mRNA in lung tissues quantified by qPCR. (**F**) H&E staining of lung tissues post-challenge. Blue dashed boxes indicate the regions shown at higher magnification in the right panels. PBS-treated mice show severe alveolar destruction and inflammatory infiltration, while immunized mice exhibit reduced pulmonary inflammation.

To evaluate the protective efficacy following immunization with the recombinant SVP candidates, a systematic analysis of mouse sera and lung tissues was performed. Serum IFA results demonstrated that no specific antibodies against HBsAg or RSV G CCD were detected in the PBS group. In contrast, mice in both the SVP-C and SVP-Y groups elicited detectable levels of anti-HBsAg and anti-RSV G CCD antibodies, with titers primarily ranging from 1:200 to 1:800 (Figure 5A). The antibody levels induced by the two SVP sources were overall comparable.

To determine whether the vaccine-induced antibodies possess neutralizing activity, an RSV neutralization assay was performed using RSV-GFP. Sera from both SVP-C and SVP-Y immunized groups markedly reduced the number of GFP-positive cells compared to the PBS control group, demonstrating that the antibodies induced by the recombinant SVPs exhibit neutralizing activity against RSV (Figure 5B). Following the RSV challenge, viral levels in lung tissues were assessed. Western blot analysis revealed strong RSV F protein signals in the lung tissues of the PBS group, whereas the levels of RSV F protein were markedly reduced in both the SVP-C and SVP-Y groups (Figure 5C). Consistently, qPCR analysis showed that the RSV F mRNA levels in the two SVP-immunized groups were significantly lower than those in the PBS group, with the Yeast-derived SVP group exhibiting a more pronounced reduction (Figure 5E).

Gross observation of the lung tissues post-challenge revealed significant hyperemia in the PBS group, along with an increased lung volume, most likely due to inflammatory responses. In contrast, the lung tissues from the SVP-C and SVP-Y groups were noticeably smaller in size (Figure 5D). H&E staining results were consistent with these findings; the PBS group showed significant alveolar structural destruction and inflammatory cell infiltration, whereas the lung tissues of both SVP-immunized groups remained relatively intact with a reduced degree of inflammation (Figure 5F).

In summary, immunization with recombinant HBsAg-RSV G CCD SVPs induces specific antibodies against both HBsAg and RSV G CCD, reduces viral loads in the lungs following RSV infection, and alleviates lung tissue damage.

## 4. Discussion

In this study, we successfully developed and evaluated an RSV G CCD recombinant vaccine candidate based on the HBsAg SVP platform. Our findings demonstrate that the HBsAg-RSV G CCD recombinant protein can be stably expressed in both mammalian and yeast cells, where it self-assembles into structurally homogeneous SVPs. Immunization studies in mice further revealed that this candidate vaccine elicited significant antibody responses and markedly reduced viral loads in the lungs following RSV challenge, highlighting the potential of this platform for generating effective RSV vaccines.

The decision to employ VLPs as the delivery platform was predicated on their inherent advantages over soluble monomeric proteins [31]. By displaying antigens in a highly repetitive spatial array, VLPs effectively cross-link B cell receptors (BCRs), thereby inducing a robust and sustained humoral immune response [32]. Furthermore, their uniform particle size of 20–25 nm facilitates direct lymphatic drainage into lymph nodes, where they are efficiently captured and presented by dendritic cells (DCs), leading to enhanced immune activation [33]. This structural configuration, which closely mimics the native architecture of viruses, likely accounts for the significant protective efficacy observed in this study.

Further investigation identified the HBsAg38 variant as superior in expression and secretion efficiency, primarily due to two amino acid substitutions—S3 and I126. These findings suggest that protein engineering strategies, such as saturation mutagenesis based on the HBsAg38 sequence, could further improve secretion efficiency and vaccine immunogenicity.

RSV G protein, as an essential target during RSV infection, offers considerable immune protection potential. While current RSV F protein vaccines have shown some success, their protective efficacy remains insufficient, particularly among infants and the elderly [10,11,12]. Real-world and clinical evidence suggest that the protective efficacy of current RSV prefusion F vaccines in older adults may wane over subsequent RSV seasons. For instance, Abrysvo’s efficacy against RSV-associated lower respiratory tract disease declined between the first and second seasons in a large phase 3 trial [15]. This highlights the limitations of the RSV F protein vaccine. RSV G CCD, being a broad neutralizing epitope, can complement the F vaccine by enhancing the production of neutralizing antibodies and potentially filling the protection gap [19,34]. Combining RSV G with the F vaccine could strengthen overall vaccine efficacy, providing broader protection against RSV.

Our study provides experimental evidence supporting the use of the RSV G protein CCD as a vaccine target. Immunization with CCD-displaying SVPs elicited antibodies that demonstrated neutralizing activity against RSV-GFP in vitro and significantly reduced viral loads and lung pathology in vivo (Figure 5). These results functionally validate the protective potential of CCD-directed immunity and support its further development as a complement to existing F protein-based vaccines.

In addition, given that hepatitis B vaccines, which use HBsAg as the primary immunogen, have been widely administered to infants, the use of HBsAg as a carrier platform for RSV G provides a safe and effective means of immunization for children [35,36]. Notably, no VED has been observed in current clinical studies of RSV G vaccines, suggesting that its use in children may not carry the same safety risks associated with other RSV vaccines [18,21,34,37]. Consistent with these findings, the immunized mice in our study exhibited no signs of VED: body weight remained stable throughout the study, H&E staining of major organs revealed no pathological changes during the immunization period, and post-RSV challenge, the lungs of vaccinated mice showed significantly reduced inflammation and tissue damage compared to unvaccinated controls, with no evidence of eosinophilic infiltration or exacerbated pathology. Nevertheless, we acknowledge that a comprehensive safety evaluation, including detailed Th1/Th2 cytokine profiling, pulmonary eosinophil quantification, and evaluation in higher animal models, will be essential to further confirm the absence of VED before clinical translation.

Finally, the flexibility of the HBsAg-SVP platform makes it not only suitable for RSV vaccine development but also offers vast potential for broader applications. Future research could integrate other pathogen-derived immunogenic epitopes into this platform, developing multivalent vaccines. For example, incorporating epitopes from the influenza virus, measles virus, or dengue virus into the HBsAg-SVP platform could leverage its strong immunogenicity and self-assembly properties to offer innovative vaccine solutions for a range of viral diseases.

Despite the strong evidence supporting the efficacy of HBsAg-RSV G CCD recombinant SVPs in mouse models, several limitations must be considered. First, our experiments were limited to mouse models, and we have yet to evaluate the vaccine in higher animal models, such as non-human primates. This is essential for assessing the vaccine safety and efficacy in a model more closely resembling human immune responses. Second, our study did not perform a comprehensive analysis of cellular immune responses. Future research should explore the role of RSV G CCD in cellular immunity to provide a more complete evaluation of its immunoprotective potential. Third, while TEM confirmed the uniform morphology and expected size range of the SVPs, more detailed particle characterization—including quantitative size distribution analysis (DLS/NTA), stability assessments, and direct quantification of surface epitope density—was not performed and warrants further investigation. Fourth, a direct comparison of antibody response magnitude with other RSV vaccine candidates was not conducted within the same experimental system. Although our in vivo challenge results confirmed the protective efficacy of the induced immune response, a comparison with other RSV vaccine platforms under standardized conditions would be a valuable direction for future studies.

## 5. Conclusions

In conclusion, we developed a recombinant vaccine candidate by inserting the RSV G protein CCD (amino acids 159–191) into a patient-derived HBsAg38 carrier, which was identified through comparative screening of clinically isolated HBsAg variants. The recombinant protein was successfully expressed in both CHO cells and *P. pastoris*, and self-assembled into uniform spherical SVPs of 20–25 nm in diameter. Safety evaluation in mice showed no detectable weight loss, organ damage, or signs of vaccine-enhanced disease throughout the study. Immunogenicity assessment demonstrated that the vaccine candidate elicited significant antibody responses against both HBsAg and the RSV G CCD, with sera exhibiting neutralizing activity against RSV in vitro. Following the RSV challenge, vaccinated mice showed significantly reduced viral loads, diminished lung inflammation, and preserved tissue architecture compared to unvaccinated controls. These results demonstrate that the HBsAg-SVP platform offers an efficient and safe approach for RSV vaccine development. With further optimization of carrier sequences and the potential incorporation of additional pathogen-derived epitopes, this platform holds great promise for developing multivalent vaccines, particularly for pediatric immunization, elderly populations, and immunocompromised individuals.

## 6. Patents

The patent related to this work is currently under application.

## Data Availability

The original contributions presented in this study are included in the article. Further inquiries can be directed to the corresponding author.

## Supplementary Materials

The following supporting information can be downloaded at https://www.mdpi.com/article/10.3390/vaccines14040311/s1.

## Abbreviations

The following abbreviations are used in this manuscript:

BCR	B Cell Receptor
BMGY	Buffered Glycerol-Complex Medium
BMMY	Buffered Methanol-Complex Medium
CCD	Conserved Central Domain
CPE	Cytopathic Effects
DC	Dendritic Cell
DMEM	Dulbecco’s Modified Eagle’s Medium
ECL	Enhanced Chemiluminescence
ELISA	Enzyme-Linked Immunosorbent Assay
FBS	Fetal Bovine Serum
FI-RSV	Formalin-Inactivated Respiratory Syncytial Virus
H&E	Hematoxylin and Eosin
HBsAg	Hepatitis B Surface Antigen
HBV	Hepatitis B Virus
IACUC	Institutional Animal Care and Use Committee
IFA	Indirect Immunofluorescence Assay
i.p.	Intraperitoneal
LRTI	Lower Respiratory Tract Infections
PEI	Polyethyleneimine
RSV	Respiratory Syncytial Virus
SVP	Subviral Particle
TEM	Transmission Electron Microscopy
VED	Vaccine-Enhanced Disease
VLP	Virus-Like Particle
WB	Western Blot
