# Supplementary material for: Development and Immunogenicity Evaluation of an RSV Recombinant Vaccine Displaying a Conserved Domain of RSV G
Source: Vaccines (Basel). 2026 Mar 30;14(4):311. doi: 10.3390/vaccines14040311 (PMC13119768; doi:10.3390/vaccines14040311)
Supplement: Supplementary file 1 [file vaccines-14-00311-s001.zip › vaccines-4204086-supplementary.pdf]

```
     1        10        20        30        40        50        60
38   MESTTSGFLGPLLVLQAGFFLLTRILTIPQSLDSWWTSLNFLGGAPTCPGQNSQSPTSNH
53   MENTTSGFLGPLLVLQAGFFLLTRILTIPQSLDSWWTSLNFLGGAPTCPGQNSQSPTFNH
61   MENIASGLLGPLLVLQAGFFLLTKILTIPQSLDSWWTSLNFLGGTPVCLGQNSQSQISSH
11   MENTTSGFLGPLLVLQAGFFLLTRILTIPQSLDSWWTSLNFLGGAPTCPGQNSQSPTFNH
12   MENTTSGFLGPLLVLQAGFFLLTRILTIPQSLDSWWTSLNFLGGAPTCPGQNSQSPTFNH
23   MENTTSGFLGPLLVLQAGFFLLTRILTIPQSLDSWWTSLNFLGGAPTCPGQNSQSPTFNH
24   MENTTSGFLGPLLVLQAGFFLLTRILTIPQSLDSWWTSLNFLGGAPTCPGQNSQSPTFNH
35   MENTTSGFLGPLLVLQAGFFLLTRILTIPQSLDSWWTSLNFLGGAPTCPGQNSQSPTFNH
46   MENTTSGFLGPLLVLQAGFFLLTRILTIPQSLDSWWTSLNFLGGAPTCPGQNSQSPTFNH
57   MENTTSGFLGPLLVLQAGFFLLTRILTIPQSLDSWWTSLNFLGGAPTCPGQNSQSPTFNH
68   MENTTSGFLGPLLVLQAGFFLLTRILTIPQSLDSWWTSLNFLGGAPTCPGQNSQSPTFNH
79   MENTTSGFLGPLLVLQAGFFLLTRILTIPQSLDSWWTSLNFLGGAPTCPGQNSQSPTFNH
80   MENTTSGFLGPLLVLQAGFFLLTRILTIPQSLDSWWTSLNFLGGAPTCPGQNSQSPTFNH
81   MENTTSGFLGPLLVLQAGFFLLTRILTIPQSLDSWWTSLNFLGGAPTCPGQNSQSPTFNH
82   MENTTSGFLGPLLVLQAGFFLLTRILTIPQSLDSWWTSLNFLGGAPTCPGQNSQSPTFNH

             70        80        90       100       110       120
38   SPTSCPPICPGYRWMCLRRFIIFLFILLLCLIFLLVLLDYQGMLPVCPLLPGTSTTSTGP
53   SPTSCPPICPGYRWMCLRRFIIFLFILLLCLIFLWVLLDYQGMLPVCPLLPGTSTTSTGP
61   SPTCCPPICPGYRWMCLRRFIIFLCILLLCLIFLLVLLDYQGMLPVCPLIPGSSTTSTGP
11   SPTSCPPICPGYRWMCLRRFIIFLFILLLCLIFLWVLLDYQGMLPVCPLLPGTSTTSTGP
12   SPTSCPPICPGYRWMCLRRFIIFLFILLLCLIFLWVLLDYQGMLPVCPLLPGTSTTSTGP
23   SPTSCPPICPGYRWMCLRRFIIFLFILLLCLIFLWVLLDYQGMLPVCPLLPGTSTTSTGP
24   SPTSCPPICPGYRWMCLRRFIIFLFILLLCLIFLWVLLDYQGMLPVCPLLPGTSTTSTGP
35   SPTSCPPICPGYRWMCLRRFIIFLFILLLCLIFLWVLLDYQGMLPVCPLLPGTSTTSTGP
46   SPTSCPPICPGYRWMCLRRFIIFLFILLLCLIFLWVLLDYQGMLPVCPLLPGTSTTSTGP
57   SPTSCPPICPGYRWMCLRRFIIFLFILLLCLIFLWVLLDYQGMLPVCPLLPGTSTTSTGP
68   SPTSCPPICPGYRWMCLRRFIIFLFILLLCLIFLWVLLDYQGMLPVCPLLPGTSTTSTGP
79   SPTSCPPICPGYRWMCLRRFIIFLFILLLCLIFLWVLLDYQGMLPVCPLLPGTSTTSTGP
80   SPTSCPPICPGYRWMCLRRFIIFLFILLLCLIFLWVLLDYQGMLPVCPLLPGTSTTSTGP
81   SPTSCPPICPGYRWMCLRRFIIFLFILLLCLIFLWVLLDYQGMLPVCPLLPGTSTTSTGP
82   SPTSCPPICPGYRWMCLRRFIIFLFILLLCLIFLWVLLDYQGMLPVCPLLPGTSTTSTGP

            130       140       150       160       170       180
38   CKTCTIPAQGTSMFPSCCCTKPSDGNCTCIPIPSSWAFARFLWEWASVRFSWLSLLVPFV
53   CKTCTTPAQGTSMFPSCCCTKPSDGNCTCIPIPSSWAFARFLWEWASVRFSWLSLLVPFV
61   CKTCTTPAQGTSMFPSCCCTKPTDGNCTCIPIPSSWAFAKYLWEWASVRFSWLSLLVPFV
11   CKTCTTPAQGTSMFPSCCCTKPSDGNCTCIPIPSSWAFARFLWEWASVRFSWLSLLVPFV
12   CKTCTTPAQGTSMFPSCCCTKPSDGNCTCIPIPSSWAFARFLWEWASVRFSWLSLLVPFV
23   CKTCTTPAQGTSMFPSCCCTKPSDGNCTCIPIPSSWAFARFLWEWASVRFSWLSLLVPFV
24   CKTCTTPAQGTSMFPSCCCTKPSDGNCTCIPIPSSWAFARFLWEWASVRFSWLSLLVPFV
35   CKTCTTPAQGTSMFPSCCCTKPSDGNCTCIPIPSSWAFARFLWEWASVRFSWLSLLVPFV
46   CKTCTTPAQGTSMFPSCCCTKPSDGNCTCIPIPSSWAFARFLWEWASVRFSWLSLLVPFV
57   CKTCTTPAQGTSMFPSCCCTKPSDGNCTCIPIPSSWAFARFLWEWASVRFSWLSLLVPFV
68   CKTCTTPAQGTSMFPSCCCTKPSDGNCTCIPIPSSWAFARFLWEWASVRFSWLSLLVPFV
79   CKTCTTPAQGTSMFPSCCCTKPSDGNCTCIPIPSSWAFARFLWEWASVRFSWLSLLVPFV
80   CKTCTTPAQGTSMFPSCCCTKPSDGNCTCIPIPSSWAFARFLWEWASVRFSWLSLLVPFV
81   CKTCTTPAQGTSMFPSCCCTKPSDGNCTCIPIPSSWAFARFLWEWASVRFSWLSLLVPFV
82   CKTCTTPAQGTSMFPSCCCTKPSDGNCTCIPIPSSWAFARFLWEWASVRFSWLSLLVPFV

            190       200       210       220
38   QWFVGLSPTVWLSVIWMMWYWGPSLYNILSPFLPLLPIFFCLWVYI....
53   QWFVGLSPTVWLSVIWMMWYWGPSLYNILSPFLPLLPIFFCLWVYI....
61   QWFVGLSPTVWLSVIWMMWYWGPSLYNILSPFMPLLPIFFCLWVYI....
11   QWFVGLSPTVWLSVIWMMWYWGPSLYNILSPFLPLLPIFFCLWV......
12   QWFVGLSPTVWLSVIWMMWYWGPSLYNILSPFLPLLPIFFCLWVYIN...
23   QWFVGLSPTVWLSVIWMMWYWGPSLYNILSPFLPLLPIFFCLWVYI....
24   QWFVGLSPTVWLSVIWMMWYWGPSLYNILSPFLPLLPIFFCLWVYIK...
35   QWFVGLSPTVWLSVIWMMWYWGPSLYNILSPFLPLLPIFFCLWVYIK...
46   QWFVGLSPTVWLSVIWMMWYWGPSLYNILSPFLPLLPIFFCLWVYIN...
57   QWFVGLSPTVWLSVIWMMWYWGPSLYNILSPFLPLLPIFFCLWV......
68   QWFVGLSPTVWLSVIWMMWYWGPSLYNILSPFLPLLPIFFCLWVYIYFQV
79   QWFVGLSPTVWLSVIWMMWYWGPSLYNILSPFLPLLPIFFCLWVYIYSQV
80   QWFVGLSPTVWLSVIWMMWYWGPSLYNILSPFLPLLPIFFCLWVYI....
81   QWFVGLSPTVWLSVIWMMWYWGPSLYNILSPFLPLLPIFFCLWVYIN...
82   QWFVGLSPTVWLSVIWMMWYWGPSLYNILSPFLPLLPIFFCLWVYIK...
```
